# A Review of the Potential of Nuclear Factor [Erythroid-Derived 2]-like 2 Activation in Autoimmune Diseases

**DOI:** 10.3390/brainsci13111532

**Published:** 2023-10-30

**Authors:** Ilker Ates, Ayşe Didem Yılmaz, Brigitta Buttari, Marzia Arese, Luciano Saso, Sibel Suzen

**Affiliations:** 1 Department of Pharmaceutical Toxicology, Faculty of Pharmacy, Ankara University, Degol Str. No. 4, 06560 Ankara, Turkey; 2 Department of Pharmaceutical Chemistry, Faculty of Pharmacy, Ankara University, Degol Str. No. 4, 06560 Ankara, Turkey; aysedidemyilmaz@gmail.com (A.D.Y.); sibel.suzen@pharmacy.ankara.edu.tr (S.S.); 3 Department of Cardiovascular and Endocrine-Metabolic Diseases and Aging, Italian National Institute of Health, 00161 Rome, Italy; brigitta.buttari@iss.it; 4 Department of Biochemical Sciences “A. Rossi Fanelli”, Sapienza University of Rome, Piazzae Aldo Moro 5, 00185 Rome, Italy; marzia.arese@uniroma1.it; 5 Department of Physiology and Pharmacology ‘‘Vittorio Erspamer”, Sapienza University of Rome, Piazzale Aldo Moro 5, 00185 Rome, Italy; luciano.saso@uniroma1.it

**Keywords:** Nrf2 activation, autoimmune diseases, inflammation, autoimmunity, immunoregulatory

## Abstract

An autoimmune disease is the consequence of the immune system attacking healthy cells, tissues, and organs by mistake instead of protecting them. Inflammation and oxidative stress (OS) are well-recognized processes occurring in association with acute or chronic impairment of cell homeostasis. The transcription factor Nrf2 (nuclear factor [erythroid-derived 2]-like 2) is of major importance as the defense instrument against OS and alters anti-inflammatory activities related to different pathological states. Researchers have described Nrf2 as a significant regulator of innate immunity. Growing indications suggest that the Nrf2 signaling pathway is deregulated in numerous diseases, including autoimmune disorders. The advantageous outcome of the pharmacological activation of Nrf2 is an essential part of Nrf2-based chemoprevention and intervention in other chronic illnesses, such as neurodegeneration, cardiovascular disease, autoimmune diseases, and chronic kidney and liver disease. Nevertheless, a growing number of investigations have indicated that Nrf2 is already elevated in specific cancer and disease steps, suggesting that the pharmacological agents developed to mitigate the potentially destructive or transformative results associated with the protracted activation of Nrf2 should also be evaluated. The activators of Nrf2 have revealed an improvement in the progress of OS-associated diseases, resulting in immunoregulatory and anti-inflammatory activities; by contrast, the depletion of Nrf2 worsens disease progression. These data strengthen the growing attention to the biological properties of Nrf2 and its possible healing power on diseases. The evidence supporting a correlation between Nrf2 signaling and the most common autoimmune diseases is reviewed here. We focus on the aspects related to the possible effect of Nrf2 activation in ameliorating pathologic conditions based on the role of this regulator of antioxidant genes in the control of inflammation and OS, which are processes related to the progression of autoimmune diseases. Finally, the possibility of Nrf2 activation as a new drug development strategy to target pathogenesis is proposed.

## 1. Introduction

Under normal conditions, the immune system protects and guards the body against attacks and infections, which are possible sources of diseases and syndromes. However, if the complex immune system regulatory network breaks down, it misguidedly attacks healthy cells, tissues, and organs. The malfunctioning of the immune system may result in attacks on any part of the body, weakening bodily functions and sometimes turning life-threatening [1]. Oxidative stress (OS) is described as a modification in the redox state [2] and the production of free radicals in cells in response to an environment of inherited traits. The immunological progression in cells may promote OS and, furthermore, the accumulation of OS worsens the pathophysiology of the disorder [3]. The over-creation of both reactive oxygen (ROS) and nitrogen (RNS) species has been associated with numerous autoimmune disorders [4]. Among the enzymes in human cells that are mainly involved in the production/bioavailability of radical species, those that are particularly relevant are NADPH oxidases (NOXs), mitochondrial electron transport chain complexes, nitric oxide synthases (NOSs), xhantine oxidases (XOs), and the hydrogen sulfide-producing enzymes cystathionine-β-synthase and cystathionine-γ-lyase [5]. ROS can actively influence both innate and adaptive immunity by regulating the response of its components at different levels, such as antigen production and apoptotic cell clearance [6]. The chemical and post-translational alterations of proteins that occur in a pro-oxidant environment may occasionally promote the formation of “neoepitopes”, which are recognized as “foreign” by the immune system and trigger the development of autoantibodies, which are made against substances formed by a person’s own body. 

**Figure 1:**
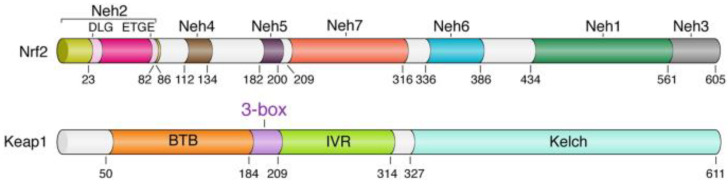
Domain architecture of the Keap1 and Nrf2 proteins. Domain boundaries and residue numbers are shown for human proteins [10].

The Kelch-like ECH-associated protein 1–nuclear factor (erythroid-derived 2)-like 2 (KEAP1-Nrf2) structure has a significant role in antioxidant reactions, and associates to protect cells from numerous redox instabilities [7,8]. The Nrf2 protein in humans is 605 amino acids long and possesses seven highly conserved areas known as Nrf2-ECH homology (Neh) domains (Figure 1). Neh1 includes the CNC–bZIP domain, which moderates heterodimerization with Maf [9]. The Neh2 domain has two degrons that are, in particular, attached by Keap1. 

Normally, Nrf2 is controlled by KEAP1. Once cells are affected by antioxidants, Nrf2 migrates into the nucleus and increases the expression of many of the genes that encode antioxidant proteins and detoxifying enzymes [11]. Nrf2 is known to be a fundamental controller of cellular defense, inflammation, and redox balance renewal [12]. Nrf2 controls the expression of over 200 genes, which are included in the promoter area called the antioxidant response element (ARE). The genes controlled by Nrf2 encode enzymes that contribute to endobiotic/xenobiotic metabolism, OS/inflammatory reactions, carbohydrate/lipid metabolism, and protein degradation [13,14].

In the course of OS, ROS interacts with the cysteine amino acid on Keap1, which modifies the formation of the Keap1–Nrf2 compound and, thus, escapes the degradation of Nrf2 [15]. For this reason, freshly gathered Nrf2 proteins gather in the cytoplasm and are then transferred to the nucleus, where the Nrf2 binds to the ARE. This supports the transcription of numerous target genes, like the detoxifying enzymes heme oxygenase (Hmox1), NAD(P)H dehydrogenase 1 (Nqo1), and sulfiredoxin 1 (Srxn1); the xenobiotic metabolizing enzymes; as well as the genes taking part in glutathione (GSH) production, such as glutathione reductase (GSR) and glutamate–cysteine ligase modifier (GCLM) [16].

**Figure 2:**
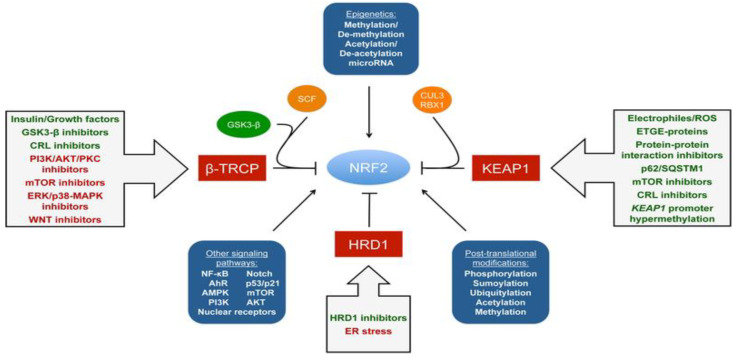
Regulation of NRF2 and possible modes of activation [18].

The regulation of Nrf2 primarily occurs through the managed maintenance of Nrf2 protein levels. There are three E3 ubiquitin ligase complexes that are responsible for the ubiquitylation and degradation of Nrf2: the CUL3-RBX1-KEAP1 complex, the SCF/β-TrCP complex, and HRD1. Each of them mediates the degradation of Nrf2 in response to various stimuli in specific subcellular compartments. The CUL3-RBX1-KEAP1 complex responds to electrophilic/oxidative stress in the cytosol. The nuclear or cytosolic SCF/β-TrCP complex is more susceptible to metabolic shifts and is regulated by GSK3-β. HRD1 is localized to the ER and has only been shown to ubquitylate Nrf2 during ER stress [17]. It is essential to state that other signaling paths, epigenetic factors, and post-translational modifications also regulate Nrf2. Similarly, the activation or inhibition of the Nrf2 path can be achieved by targeting the negative regulation of Nrf2 (Figure 2).

The molecular activation and cytoprotective activity of the KEAP1-NRF2 route comprise four separate but interlinked elements:

(a) The chemical inducers of NRF2 activity (e.g., tBHQ, CDDO-Im, and TBE-31 [19]) (Table 1);
(b) KEAP1, the protein sensor of these inducers;
(c) The transcription factor NRF2, which regulates the transcriptional response to inducers and oxidative stress;
(d) The target genes that supply the cytoprotective output of the path [20].

**Figure 3:**
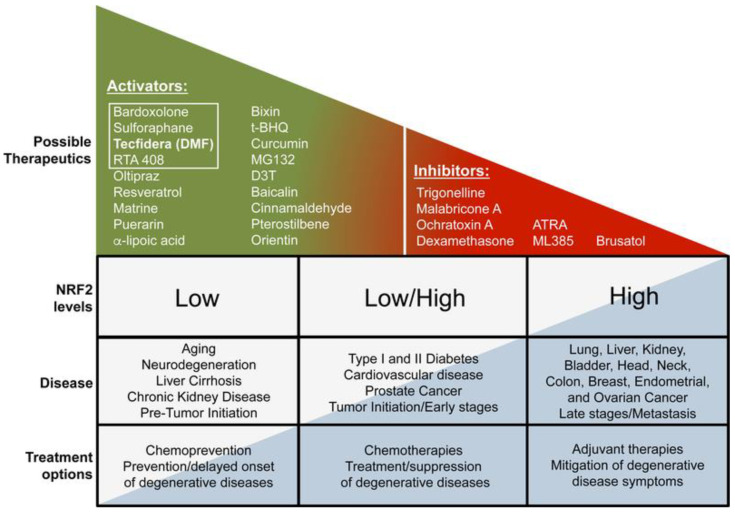
Nrf2-targeted therapeutics [18].

There is a developing theme in the Nrf2 area that targeting Nrf2 in disorder is both context and time-dependent. Harnessing the advantageous outcomes of pharmacological activation of Nrf2 is an essential part of Nrf2-based chemoprevention and intervention in other chronic illnesses, such as neurodegeneration, diabetes, cardiovascular disease, autoimmune diseases, and chronic kidney and liver disease. Nevertheless, a growing number of investigations have indicated that Nrf2 is already elevated in specific cancer and disease steps, suggesting that pharmacological agents developed to mitigate the potentially destructive or transformative results associated with protracted activation of Nrf2 should also be considered. Instances of current Nrf2 activators and inhibitors, as well as Nrf2 expression grades in disorders, are outlined in Figure 3 [18]. Nrf2-targeted therapeutics are shown in Table 2.

The cellular protection ability supported by Nrf2 activation seems to maintain the stability of CD4^+^ and CD8^+^ of lymph node cells for appropriate essential immune reactions. Nrf2 is able to able to negatively regulate pro-inflammatory signaling molecules like p38 MAPK, NF-kB, and AP-1. Another task of Nrf2 is to prevent the formation of pro-inflammatory intermediates such as cytokines, chemokines, cell adhesion molecules, COX-2, matrix metalloproteinases, and iNOS. Even though not openly clarified, the antioxidant activity of genes directed by Nrf2 might supportively control the innate immune defense [22]. 

It seems that Nrf2 action is crucial in regulating cellular mechanisms regarding the determination of inflammatory progression. To accomplish this, Nrf2 interacts with nuclear factor-κB (NF-κB) via various chemical connections [23]. The phosphorylation reaction of NF-κB inhibitor (IκBα) by IκB kinase (IKKβ) leads to IκBα deprivation, which causes nuclear translocation and DNA attachment to NF-κB. Nrf2 reduces NF-κB stimulation by reacting with Keap1. Nrf2 is displayed to extensively regulate the immune defense by acting with vital innate immune elements such as the toll-like receptors–Nuclear factor kappa B (NF-κB) cascade, inflammasome signaling, and the type-I interferon reaction. 

The role of Nrf2 in downgrading inflammation has been recognized in some animal studies with different pathological disorders [24]. Furthermore, Nrf2 absence worsens autoimmune disorders like rheumatoid arthritis (RA) [25] and systemic lupus erythematosus (SLE) [26], whereas Nrf2 activation improves autoimmune encephalomyelitis [27].

**Figure 4:**
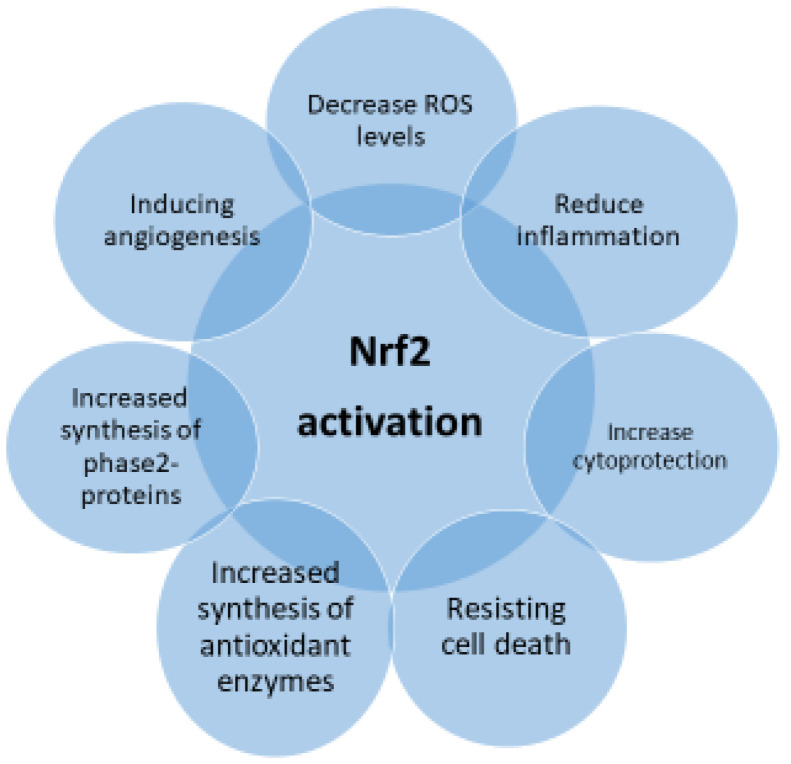
Pharmacological properties of Nrf2 activation.

Growing indications have reinforced the idea that the regulatory role of Nrf2 is not limited to OS but is also directed to inflammation mechanisms, immune system disorders, and cartilage and bone metabolism [28]. Research has shown that Nrf2 activation might regulate diverse progressions and intermediates that take place in the pathology of autoimmune disorders such as RA, systemic SLE, osteoarthritis (OA), and osteoporosis. Nrf2 activation, through multiple pathways, induces a strong antioxidant response associated with beneficial effects against various conditions (Figure 4). The goal of this observation is to emphasize Nrf2 as a novel pharmacological target in above mentioned illnesses.

## 2. Nrf2 Regulation and Inflammation

**Figure 5:**
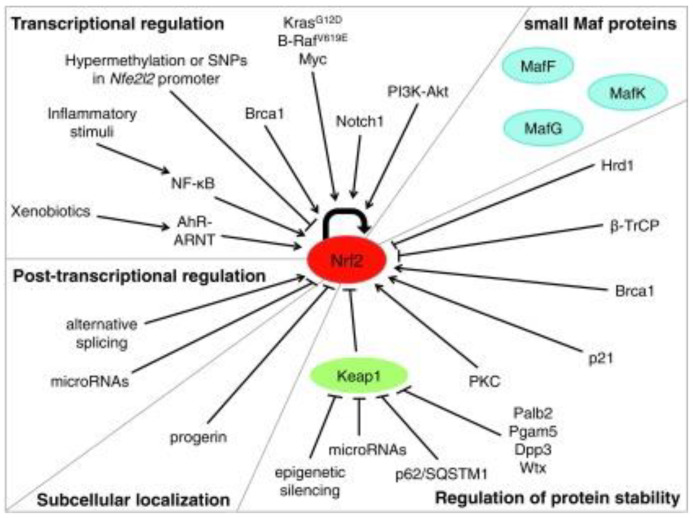
Mechanisms of regulation of Nrf2 activity [29].

Given the extensive display of stimuli that trigger Nrf2 and the various cellular operations that it handles, the regulation of Nrf2 activity is complicated and multifactorial. Absolutely, Nrf2 activation can be managed at the transcriptional and post-transcriptional level via the regulation of protein stability, post-transcriptional shifts, and the availability of binding fellows (Figure 5).

Inflammation is the most known consequence of numerous immune disorders and illnesses. Research has shown that Nrf2 is linked to anti-inflammatory progression by altering the employment of inflammatory cells and by modifying gene expression at the ARE level. The Nrf2 protein half-life is less than 20 min in the cell [30]. The Keap1/Nrf2/ARE signaling pathway primarily controls anti-inflammatory gene expression and prevents the development of inflammation in many diseases [31]. Other signaling pathways like NF-κB, MAPK (mitogen-activated protein kinase), and JAK (janus kinase)-STAT (signal transducers and activators of transcription) are considered to be of relevance in the progress of inflammation [32]. It was discovered that Nrf2 controls the phase II detoxifying enzymes involving NADPH, NAD(P)H quinone oxidoreductase 1, glutathione peroxidase, ferritin, heme oxygenase-1 (HO-1), and antioxidant genes that protect tissues against damage with their anti-inflammatory properties [33].

The main role of inflammation is to eliminate the foundation of disorder and re-establish homeostasis. Conventionally, native immunity is the prompt reaction in the procedure of phagocytosis, while adaptive immunity is antigen-reliant and described by an immunological recall that allows for increasing an effective immune response following contact with a similar antigen. The symbol of the inflammatory process is the formation of signaling elements named cytokines [34]. Activation of Nrf2 in several diseases related to OS and inflammation lessens markers of damage and inhibits illness development. These properties are thought to be the result of the upregulation of antioxidative and phase II detoxifying enzymes by Nrf2 as well as the straight role of control in the formation of inflammation. It is known that Nrf2 prevents transcription of pro-inflammatory IL-6 and IL-1β cytokines by making bonds to genes in macrophages and stopping RNA Pol II employment [12].

**Figure 6:**
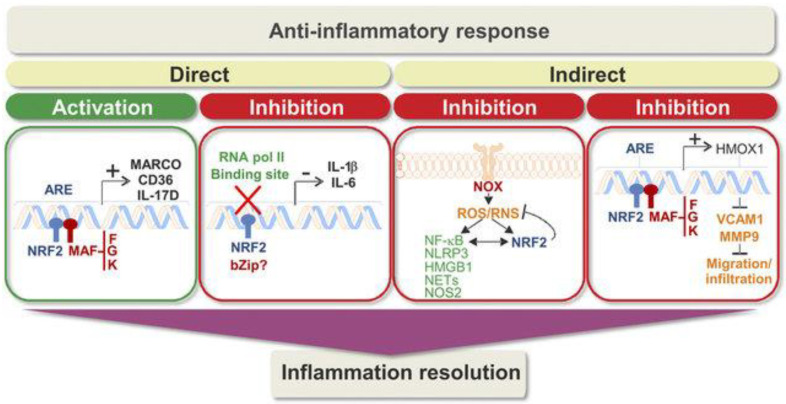
Direct and indirect regulation of inflammation by Nrf2 [40].

Because Nrf2 is a lead regulator of redox homeostasis, it wields indirect control on NF-kB activity. Lipopolysaccharides (LPSs) trigger simultaneously a fast, pro-inflammatory NF-kB response and a slow Nrf2 reaction. The NF-kB response is subsequently inhibited while Nrf2 is maximally functional [35]. For example, Ras-related C3 botulinum toxin substrate 1, a small G protein of the Rho family, activated the NF-kB path and Nrf2 overexpression obstructed, whereas Nrf2 knockdown improved NF-kB-dependent transcription [35]. Invariably, in Nrf2-deficient (Nrf22/2) mice challenged with LPS or tumor necrosis factor (TNF)-a, the activity of IKK was aggravated and led to raised phosphorylation and degradation of IkB [36]. Nrf2 furthermore generates an anti-inflammatory phenotype that modulates the functions of CD8+ T cells [37] as well as in macrophages and microglia [38,39,40]. This is because Nrf2 augments cysteine and GSH levels in macrophages via regulation of the cystine/glutamate transporter and the GSH-synthesizing enzyme g-glutamyl cysteine ligase modulator and catalytic subunits (g-glutamyl cysteine ligase modulator subunit (GCLM) and g-glutamyl cysteine ligase catalytic subunit (GCLC)). Contrarily, a lack of GSH sensitizes macrophages to Nrf2 activation by LPS [41]. All these investigations indicate Nrf2 as an anti-inflammatory factor critical in maintaining the intensity and period of inflammatory reactions (Figure 6).

Direct mechanisms of activity contain transcriptional induction of anti-inflammatory genes as well as transcriptional repression of pro-inflammatory genes. In the second box, the question mark reveals that additional work is needed to determine the bZip partner of Nrf2 in this function, if any. Indirect mechanisms to compensate for inflammation include ROS/RNS modulation and inhibition of the migration/infiltration of immune cells. In general, these paths guide an anti-inflammatory reaction that properly resolves inflammation. The presence of polymorphisms in NFE2L2 related to diminished transcriptional activity, the varied levels of target genes in patients, and promising data from preclinical analyses support a suitable position of Nrf2 in inflammation resolution [42].

Carbon monoxide (CO) is a steady gaseous molecule that reacts selectively with transition metals in a distinct redox state, and these characteristics limit the interaction of CO with described biological targets that transduce its signaling action [43]. Because of the increased affinity of CO for ferrous heme, these targets can be categorized into heme-containing proteins, illustrating an enormous assortment of sensors and enzymes with a sequence of various roles in cells and organisms. Despite this concept, advancement in specifying which of these targets are selective for CO has been slow, and even the significance of raised carbonmonoxy hemoglobin, a classical marker utilized to diagnose CO poisoning, is not well-comprehended. Nonetheless, the usage of CO gas and CO-releasing molecules as pharmacological strategies in models of disease has supplied new vital knowledge about the signaling properties of CO. CO is continually yielded by heme oxygenases in mammalian cells during heme degradation [44].

Heme oxygenases exist in constitutive (HO-2) and inducible (HO-1) isoforms and are derivatives of two different genes, HMOX2 and HMOX1. While heme oxygenase-2 (HO-2) has separate tissue localization, being predominantly expressed in the testes, brain, and endothelium, heme oxygenase-1 (HO-1) is upregulated in all tissues investigated, pursuing several types of stress stimuli involving oxidative stress, which is an underlying factor in various pathological conditions [44].

HO-1, also known as heat shock protein 32, is the rate-limiting and inducible cytoprotective enzyme in the heme degradation path that degrades heme into free iron, carbon monoxide, and biliverdin, which is then rapidly converted into bilirubin [45,46]. Biliverdin is henceforward reduced to bilirubin via biliverdin reductase. Both biliverdin and bilirubin are bile pigments with antioxidant effects [47]. Endogenous CO can function as a second messenger, thereby affecting a variety of physiological and pathological processes involving cell proliferation, inflammation, apoptosis, and injury [48]. As a cytoprotective enzyme, HO-1 serves an essential function in controlling cell homeostasis.

Under miscellaneous pathophysiological stress or stimulation conditions, such as hypoxia, ultraviolet light, inflammatory mediators, heme, ischemia, and other harmful stimuli, HO-1 expression is generated to guard cells against oxidative and inflammatory harm [49]. In the presence of the forenamed stimulatory elements, the yielded expression of HO-1 is mainly influenced by redox-sensitive transcription factors, involving Nrf2, activator protein 1 (AP1), hypoxia-inducible factor (HIF), and BTB and CNC homology 1 (Bach1) [50].

The association between Nrf2 and the induction of HO-1 is well established and is conditional on the existence of antioxidant reaction elements in the promoter of the HMOX1 gene [51,52].

Nrf2 is now known as the lead regulator of cellular antioxidant protection systems because it handles, in addition to HO-1, the expression of a battery of detoxification enzymes, such as NAD(P)H dehydrogenase quinone 1, glutathione S-transferases, and peroxiredoxins [53,54,55]. Therefore, the defense wielded by Nrf2 is reliant on these genes, and their silencing, to a significant capacity, switches the helpful actions of Nrf2 activation [56]. It is not surprising then, that many of the HO-1 inducers that have been represented by various authors over the last decade seem to include Nrf2 as the upstream factor inducing this response [57].

It is also fascinating to report that the transcription factor Nrf2 was significantly augmented for the genes positively associated with HMOX1. The Nrf2-Keap1-HMOX1 path is a cellular defense mechanism playing a vital role in shielding against oxidative stress and inflammation [58]. This pathway is activated in response to diverse stimuli, involving reactive oxygen species (ROS), heavy metals, and xenobiotics. Once triggered, it leads to the transcriptional upregulation of antioxidant and detoxifying genes involving HMOX1 [59,60].

Mangano et al. [61] designed a study to explore the immunoregulatory mechanisms operating in the development and regulation of concanavalin A (ConA)-induced hepatitis. They evaluated the role of the anti-inflammatory path Nrf2/HO-1/CO in this condition and investigated the in vivo administration of CO through the CO-releasing molecule (CORM). They observed that the Nrf2/HO-1/CO pathway is fundamental for immune response regulation. Also, Nikolic et al. [62] tried to find efficacy and the mechanisms of action of the CO-releasing molecule (CORM)-A1 in preclinical models of type 1 diabetes. Their data indicated that the capability of CORM-A1 to save mice from developing type 1 diabetes supplies useful evidence of conception for the probable exploitation of controlled CO delivery in clinical settings for the therapy of autoimmune diabetes.

In the absence of Nrf2, oxidative cell injury and apoptosis may increase the formation of autoantigens, leading to the triggering of T cells and the creation of autoantibodies by B cells. Furthermore, the lack of phase II enzymes results in an increase in ROS. Nrf2 is a chief controller of cellular defense reactions to oxidation, and it is expected that Nrf2 activation is able to defend against OS associated with autoimmune pathogenesis [27,28].

Studies propose that Nrf2 responds to the NF-κB-associated inflammation reaction by challenging the transcription co-activator cAMP response element (CREB) binding protein (CBP) [63]. The histone acetylation reaction and subsequent DNA transcription are controlled by CBP-p300. CBP was stated to interrelate with the domains Neh4 and Neh5 of Nrf2, causing the acetylation of Neh1, which is directly involved in DNA binding [64]. It was described that the link between the N-terminal area of the p65 subunit of NF-κB and Keap1 could stop the Nrf-2 cascade. Nevertheless, various studies reported that diverse causes of OS usually stimulate both NF-κB and Nrf2-ARE signaling [65,66].

Inflammation is a multifaceted interaction among several inflammatory cells, which gives many signaling agents like arachidonic acid type compounds, phospholipid mediators, and cytokines that seem to have an essential part in some inflammatory responses, influencing the reactions between pro- and anti-inflammatory systems that lead to various disorders [67]. The activation of the Nrf2 pathway determines a remarkable event to address and direct the evolution of inflammation. It was described that the activation of Nrf2 inhibits LPS-induced modulation of pro-inflammatory cytokines including IL-6 and IL-1β. This relationship was confirmed by the finding that Nrf2 controls NF-κB-oriented transcription of pro-inflammatory cytokine genes [11].

Mills et al. [68] described the effects of a novel itaconate derivative, 4-octyl itaconate, that protects mortality in vivo and reduces cytokine formation. They showed that type I interferons increase the expression of Irg1 (also known as Acod1) and itaconate formation. In addition, they found that this reaction confines the type I interferon response. Their data show that itaconate is an important anti-inflammatory molecule that limits inflammation through Nrf2 and modulates type I interferons.

Yan et al. [69] found that treatment with dimethyl fumarate considerably enhanced cognitive insufficiencies and partly inverted neuronal injury in the hippocampus triggered by chronic cerebral hypoperfusion (CCH). In addition, this management reduced the concentration of the pro-inflammatory cytokines IL-1β, TNF-α, and IL-6 in the hippocampus and mediated NF-κB signaling. The results suggest that dimethyl fumarate may increase cognitive injury in rats with CCH, undoubtedly by lessening inflammation and ferroptosis of neurons.

Ding et al. [70] reported that Nrf2 absence considerably raised IL6 and IL10 secretion by M1 macrophages. The control of these macrophage inflammations via Nrf2 shows numerous roles for Nrf2 in modifying inflammation in macrophages. A lack of Nrf2 augmented the Glu4 protein level and reduced AKT and GSK3β protein phosphorylation in M1 macrophages, signifying multiple roles for Nrf2 in modifying glucose metabolism in macrophages. These results back the perspective that Nrf2 is a pharmacological target for the inhibition and cure of inflammation and obesity-linked disorders such as diabetes and atherosclerosis. 

Numerous in vivo and in vitro experiments have revealed the result of Nrf2 in retreating diabetes mellitus by responding to the progression of OS [71]. Nrf2 expression has been found to be induced under OS conditions. Therefore, there is an urgent need for research and clinical trials to develop essential therapeutics to protect against the progression of diabetes and to upregulate genes contained in Nrf2 as a means of combating hyperglycemia [72]. In addition, Nrf2 functions as a chief factor in detoxifying cellular reactions that deliver sufficient defense against OS and damage. Several lines of evidence point to the vital act of OS in diabetes. A similar theory also shows that an increase in ROS significantly causes the growing link between free fatty acids and hyperglycemia. Furthermore, ROS activates stress-sensitive signaling pathways, ultimately leading to diabetes mellitus, β-cell dysfunction, and insulin resistance [73,74].

As mentioned above, the upregulation of specific Nrf2 target proteins such as glutathione S-transferase, glutamyl cysteine synthase, quinone oxidoreductase, and heme oxygenase-1, takes place through specific elements that represent an antioxidant reaction and are located in the promoters of these genes. This synchronized act of modified genes encoding antioxidant, anti-inflammatory, and detoxifying regulators functions as possible healer compounds to protect against the increased OS and inflammation in diabetes mellitus [75]. 

Ferroptosis is a type of cell death mechanism that takes place intracellularly in the presence of iron. This system is a different action from cell apoptosis, necrosis, and autophagy, and it is defined by an imbalanced redox system and augmented amounts of intracellular ROS [76]. Li et al. [77] showed that ferroptosis is involved in the development of diabetic nephropathy, which is possibly a consequence of reactions between metabolic and hemodynamic mechanisms. It seems that the metabolic and hemodynamic disorders found in diabetes interrelate with ROS formation. Gene regulation and stimulation of transcription factors are affected by contacts between metabolic inducements, hemodynamic issues, and several ROS in diabetes [78]. The upregulation of Nrf2 by fenofibrate treatment inhibited diabetes-related ferroptosis and delayed the progression of diabetic nephropathy. Li and his colleagues’ research revealed the mechanism of the development of diabetic nephropathy from a new perspective and provided a new approach to delaying the progression of diabetic nephropathy.

There are several reports on the positive effects of sulforaphane, extracted from broccoli sprouts, on macrovascular complications in diabetes [79,80,81,82,83]. Dh404 is a bardoxolone methyl derivative that has been used clinically for the management of diabetic nephropathy. Dh404 triggers Nrf2 by an alteration of KEAP1, a reaction similar to sulforaphane [84]. Tan et al. [85] reported that Dh404 reduced atherosclerosis in diabetic conditions at lower doses with a reduction in OS and inflammatory factors. Dimethyl fumarate is a recognized Nrf2 activator and is used clinically for the management of multiple sclerosis. The study by Ha et al. [86] suggests a possible defensive effect of dimethyl fumarate on macrovascular complications in diabetes. Tert-butyl hydroquinone stimulates Nrf2 by directing Cys-151 in the KEAP1 protein [19]. It was shown to improve diabetes-related atherosclerosis in an animal study [87]. It was found to increase Nrf2 action in macrophages in atherosclerotic lesions and promote autophagic activity. This resulted in a reduction in atheroma plaque size, expansion, and lipid content, as well as in decreased lesional macrophages, foam cell size, and chemokine expression.

Yu et al. [88] suggested that high uric acid levels trigger ferroptosis of macrophages in the development of atherosclerosis. Additionally, promoting autophagy and preventing ferroptosis by triggering Nrf2 might ameliorate atherosclerosis caused by elevated uric acid concentrations. These results may provide a better understanding of the role of high uric acid concentrations in the development of atherosclerotic plaques and show a pharmacological approach for atherosclerotic vascular disease connected with high uric acid levels. 

In contrast, Li et al. [89] indicated that Nrf2 deficiency is associated with a reduction in atherosclerotic plaques and may reduce physiopathological development by attenuating lectin-like oxidized low-density lipoprotein receptor-1 (LOX-1)-mediated production and relocation of vascular smooth muscle cells. 

Zhao et al. [90] show that melatonin may be effective in protecting against smoking-related vascular damage and atherosclerosis through the Nrf2/ ROS /NLRP3 cascade. Generally, these findings deliver convincing support for the use of melatonin clinically to reduce inflammatory vascular damage and atherosclerosis caused by smoking.

Feng et al. [91] demonstrated the positive characteristics of kaempferol against postmenopausal atherosclerosis related to the PI3K/AKT/Nrf2 pathway facilitated by G protein-coupled oestrogen receptor (GPER) activation.

## 3. Keap1/Nrf2/ARE Signaling Pathway: Possible Link to Anti-Inflammatory and Antioxidant Mechanisms

OS may trigger a range of transcription factors such as NF-κB, AP-1activator protein 1, p53, HIF-hypoxia-inducible factor 1α, peroxisome proliferator-activated receptor γ (PPAR-γ), β-catenin/Wnt, and Nrf2. Stimulation of these features causes the expression of more than 500 altered genes. Excess amounts of ROS, with respect to the levels needed to support the cellular radical scavenging systems, could be a source of OS and trigger pro-inflammatory pathways [92]. Keap1/Nrf2/ARE controls GSH amounts by modulating GSH enzymes. Nrf2 also upregulates glutamate cysteine ligase (GCL). It was revealed that the Nrf2-Keap1 pathway controls cytosolic and mitochondrial ROS formation. A lack of Nrf2 causes improved NOX2 activity, whereas a decreased level of Keap1 leads to the maintenance of NOX4 activity. This evidently shows the vital role of Nrf2-Keap1 in redox homeostasis and NADPH oxidase [93]. In addition to the direct modification of ARE-responsive genes, Nrf2 similarly backs antioxidant and detoxification paths by supporting the formation of NADPH, which is a powerful antioxidant [94].

## 4. Diabetes Mellitus and Nrf2 Activation

Diabetes mellitus (DM) is described as a complicated metabolic disorder with hyperglycemia as one of its most known consequences. It is recognized for causing problems in the heart, kidneys, eyes, and blood vessels according to the American Diabetes Association [95]. Diabetic nephropathy (DN) is one of the primary diseases that evolve in both type 1 (T1DM) and type 2 (T2DM) diabetes mellitus [96]. The last symbolizes the most familiar diabetes type of final-stage renal condition (ESRD) [97]. The phrase diabetic kidney disease (DKD) contains both DN, with albumin defeat in the urine and renal activity damage, and a special state of nephropathy characterized by the decrease in GFR lacking albumin in urea [98,99]. Despite its multisystemic pathological condition, with numerous characteristics having an essential role, involving being elderly and having high blood glucose, high blood pressure, inflammation, metabolic syndrome, and ischemic heart disease, the detailed physiological progressions that happen are still unknown [100,101].

OS is implicated in playing a vital role in the progress of DKD [102]. The heart and kidneys are the first two locations regarding the number of mitochondria and oxygen uptake [103]. At the mitochondrial level, exaggerated ROS are developed due to hyperglycemia. This can influence the primary antioxidant protection systems [71]. Thus, directing the components of oxidation and antioxidants via the Nrf2/KEAP1/ARE system might be confirmed as an effective strategy in the treatment or management of DKD [102]. Considering the influential action of OS in the growth of DKD, with the Nrf2/KEAP1/ARE system as a principal controller of the oxidation of the cell, concentrating on this pathological unit may represent a suitable target for a deeper and tuned control of this upsetting disease [104].

Macrovascular difficulties of diabetes (MCD) include ischaemic heart conditions, cerebrovascular disorders, and peripheral vascular disease, which are developed by over half of diabetic patients, causing elevated morbidity and mortality. Stimulation of Nrf2 is valuable to the vasculature beneath diabetic situations before the formation of atherosclerotic plaques [105].

Modulation of the Nrf2-induced antioxidant response is important in the early stage of diabetes-related cardiovascular complications. Recent investigations indicated an increasing Nrf2-based approach for the treatment of diabetes, with a precise focus on the stimulation of Nrf2 by antioxidant molecules and nanoparticles. Accumulating evidence implies a prominent act for Nrf2-linked antioxidant reaction, one of the most-considered cellular protective systems against ROS increase [106].

Diabetic patients generate numerous ophthalmic difficulties involving retinopathy, corneal abnormalities, glaucoma, and cataracts. Retinopathy is one of the most recurrent and disturbing of these disorders [107]. Increased glucose stimulates numerous metabolic anomalies, and due to an inequality between the production and demolition of ROS, their levels are enhanced. The metabolic features of the retina, particularly exposure to free radical-induced damages make this tissue highly sensitive to hyperglycemia, resulting in stimulation of protein kinase C (PKC), polyol and hexosamine systems, and proliferation of progressive glycation end products (AGEs) that ultimately contribute to the evolution of retinopathy. All of these disorders are diligently linked with an increase in OS [108,109].

Dysregulation of Nrf2 has also been shown in type I and II diabetes. Augmented oxidative stress is a prevailing characteristic of diabetes that leads to cellular dysfunction and metabolic shifts in several tissues. The role of Nrf2 in diabetes is complicated and tissue/cell type-dependent. SF and cinnamic aldehyde (CA, natural, flavonoid) were both offered to repress oxidative damage and improve normal kidney function in a streptozotocin-induced mouse model of type I diabetes [72]. Oral administration of CDDO-Im resulted in enriched Nrf2 activity and attenuation of the diabetic phenotype in db/db mice [110]. CDDO-Im, CDDO-Me, oltipraz, and curcumin improved insulin sensitivity and glucose tolerance in both genetic and high fat-diet-induced diabetic models [111,112,113,114].

Interestingly, Keap1flox/- mice, which have constitutively more elevated levels of Nrf2, also displayed postponed onset of diabetes when crossed with db/db mice [110]. Yet, further studies revealed that KEAP1 knockdown improved the diabetic phenotype in Lepob/ob mice and mice fed a high-fat diet [115,116], suggesting dietary and genetic factors influencing Nrf2 may impact the onset and progression of diabetes differently. CDDO-Me was demonstrated to enrich kidney function and reduce body weight in patients with diabetic nephropathy [117]; nevertheless, the investigation was terminated due to the increased risk of cardiovascular occasions. Notably, enhanced particularity and the appropriate clinical context could still generate a positive effect for this and other Nrf2-based drugs [118].

## 5. Multiple Sclerosis and Nrf2 Activation

Multiple sclerosis (MS) is a neurologic autoimmune disorder described as chronic inflammation of the central nervous system (CNS) accompanied by demyelination and axonal impairment [119,120,121]. There is increasing proof that the pathogenesis of MS involves the unrestrained formation of ROS and reactive nitrogen species (RNS) associated with mitochondrial dysfunction and energy depletion [121,122,123]. Inflammation and OS appear to be near corresponding processes. OS induces demyelination and neurodegeneration instantly by lipid peroxidation, proteins, and DNA oxidation [124,125]. Many of the actors involved in this delicately adjusted network are maintained by the Keap1/Nrf2/ARE signaling pathway, a main regulator of antioxidant and phase II detoxification genes [120,121,126,127]. This pathway also has a vital role in the inflammation process and thus has significant potential in the treatment of MS [31].

## 6. Systemic Lupus Erythematosus and Nrf2 Activation

SLE is a complicated chronic autoimmune disorder characterized by antibodies to nuclear and cytoplasmic antigens, multisystem inflammation, protean clinical manifestations, and a relapsing and remitting course [128]. SLE reflects the complex cellular and molecular mechanisms involved in its pathogenesis. Unusual consent of apoptotic bodies tracked by autoantibody formation to nuclear antigens and damage is important for SLE progress. The immune autoantibody/antigen structures are placed in organs and tissues triggering inflammation and, subsequently, tissue injury. Due to the toxic outcomes, enhanced morbidity and mortality are observed in persons with SLE. Novel treatments containing improved effectiveness and no toxicity are required [12].

Inequality among the cellular formation of ROS and defense capability results in in the onset of OS. This OS state is aggravated by inflammation, an additional vital factor in aging and the development of chronic conditions similar to SLE [129,130]. Similarly, OS can guide to, or extend, inflammation [131].

An increasing number of studies on the mechanism of OS and inflammation suggest a theoretically important position for Nrf2 in SLE [132]. Nrf2 shortage leads to lupus-like autoimmune illness in female animals, indicating a straight role of Nrf2 defect in the pathogenesis of autoimmune disorders [133]. Furthermore, extensive DNA injury by OS, Nrf2, and the Nrf2 transcriptional target NQO1 in the glomeruli of SLE patients have possibly established a cell reaction to OS connected with SLE [134]. Therefore, Nrf2 activity in SLE could be vital for supporting redox homeostasis, typical immune reactions, and reducing cell damage. Three Nrf2 agonists have entered clinical research trials as a treatment for SLE (Table 3).

## 7. Inflammatory Bowel Disease and Nrf2 Activation

Inflammatory bowel disease (IBD), is defined as a chronic, inflammatory affection of the gastrointestinal tract, including ulcerative colitis (UC) and Crohn’s disease (CD) [135]. Even though the reason for IBD is yet not totally comprehended, it is believed that its pathogenesis includes around a hundred genetic aspects and multiple environmental reasons including smoking, nourishment, or stress [136,137,138]. Among genetic aspects that encourage IBD are genes and proteins that control OS, redox signaling, and inflammation. In such context, the Nrf2/Keap1 pathway is suggested to have a shielding outcome in individuals with IBD [139,140].

Three distinct types of intercellular junctions can be distinguished in the sheet of the intestinal epithelial cells [141]. It was demonstrated that the Nrf2/ARE stimulating action is essential to maintain TJs protein expression and membrane assemblage in the intestines of IBD patients, esophageal and alveolar epithelium [142,143,144]. The mechanisms might contain OS-mediated mtDNA modifications in guardian intestinal epithelial cells. This is a progression related to the stimulation of Nrf2/ARE signaling and the decline in mitochondrial ROS engaging in intestinal epithelial cell damage [145].

The aforementioned studies prove that the Nrf2/Keap1 signaling pathway controls GI tract activity. Research on the defensive action of Nrf2 in UC focuses on its capacity to maintain the concentration of antioxidative enzymes like HO-1 and NQO1, interleukins (IL)-6, IL-1β, and IL-17 [146,147,148], and autophagy [11,149,150]. One of the human studies that revealed the probable role of Nrf2 in UC was performed by Arisawa et al. [151]. They revealed a relationship between Nrf2 gene polymorphism and UC. An augmented Nrf2 amount is associated directly with glutathione S-transferase A4 and peroxiredoxin-1 [152]. Multiple studies reported either an increase or a decrease in Nrf2 protein levels in the mucosal tissue of IBD patients [153,154,155,156]. These findings suggested an adaptive and Nrf2-driven reaction of colonic epithelial cells to OS during chronic intestinal inflammation.

In experimental animal models of IBD, Nrf2 shortage has been revealed to boost susceptibility to colitis [157,158]. The complexity of the Nrf2/Keap1 pathway also extends to disease advancement, in which case both shortage and excess of Nrf2 have been demonstrated to deteriorate disease in animal models [159,160]. These observances highlight the dual role or hormetic nature of NRF2 in biology, which is characterized by a biphasic response to Nrf2 activation.

## 8. Autoimmune Addison’s Disease and Nrf2 Activation

Autoimmune Addison’s disease (AAD) is described by the autoimmune damage of the adrenal cortex [161,162]. The illness requires lifetime steroid hormone support treatment. Autoimmune etiology is usually evident from the company of other related autoimmune disorders [163,164]. Initially, glucocorticoids were considered an unrecommended treatment because of their capability to improve blood glucose concentration. Undoubtedly, adrenalectomized animals or people with adrenocortical insufficiency (Addison’s disease) suffer from ongoing hypoglycemia [165,166]. Notably, Nrf2 has a double act in both cancer establishment and development. At earlier stages, Nrf2 exerts a defensive role against cancer progress by inhibiting the ROS-induced mutagenic consequences of carcinogens by triggering the transcription of several cytoprotective genes responsible for GSH synthesis, redox homeostasis, xenobiotics detoxification, and anabolic biotransformation [167,168]. Nrf2 is unable to increase transcriptional activation being greatly glycated, inconsistent, and impaired in binding to small proteins in cells with musculoaponeurotic fibrosarcoma (MAF). Notably, glycation significantly improves Keap1-mediated Nrf2 degradation. Consequently, several research groups have revealed that induction of the glyoxalase detoxification system by Nrf2 signaling illustrates an important protection mechanism against decarbonyl glycation-induced pressure under circumstances of chronic hyperglycaemic, inflammation, cellular aging, and senescence by remarkably decreasing toxic levels of methylglyoxal (MGO), glyoxal (GO), and other advanced glycation end products (AGEs) through catabolization of these two AGE precursors.

## 9. Graves’ Disease and Nrf2 Activation

Graves’ disease (GD) is an immune system disease that causes the excess formation of thyroid hormones (hyperthyroidism). Even though many diseases can cause hyperthyroidism, GD is the most-known reason. Research has revealed that Nrf2 is a crucial agent in the thyroid gland. According to in vivo experiments Nrf2 supports the formation of antioxidant and cytoprotective components like Nqo1, Gpx2, and Txnrd1 in the thyroid gland [169]. Nrf2 activation by iodine is possible by physiological oxidation reactions including thyroid hormone synthesis [170]. Nrf2 controls the transcription of the gene encoding Tg by affecting AREs. In vivo studies suggest that an autoimmune response and Nrf2 signaling are part of the reaction of the thyroid to iodine and the physiopathogenesis of GD [171].

Augmented levels of OS markers are found in patients with GP, including urinary levels of the oxidized guanosine species MDA [172]. Although hypothyroidism could be treated with T4, peculiarly, GD patients need to be treated with anti-thyroid drugs. It was found that indoline and benzimidazole derivatives activate Nrf2 and are suggested as adjunct treatments for Hashimoto’s and GD [173].

## 10. Nrf2 and Rheumatoid Arthritis

Rheumatoid arthritis (RA) is a chronic autoimmune disease of unknown etiology, which affects approximately 0.5–1.0% of the world’s population. It frequently contributes to joint involvement, synovitis, and intra-articular cartilage damage [174,175]. It is believed that the etiology of RA is closely related to one’s living environment, genetics, immunity, and additional factors. People with genetic factors are impacted by their living environment, stress, and other factors, which generate abnormal reactions in the innate and adaptive immune systems, guiding the destruction of immune tolerance and thus stimulating an inflammatory response [176,177]. The main pathological characteristic of RA is inflammation leading to articular cartilage injury provoked by cartilage degradation. Many investigations have shown that Nrf2 activation is a promising method for the treatment of RA [178]. The Kelch-Nrf2/ARE signal transduction pathway can have advantageous anti-inflammatory and antioxidant effects and can regulate oxidative stress in RA. At its core, advanced Nrf2 activity can regulate mitochondrial function and restrict the production of mitochondrial ROS after activation of this pathway [179]. At present, two Nrf2 agonists have entered clinical research for rheumatoid arthritis (Table 4).

## 11. Effects of Nrf2 Modulation on Brain Health

Disorders in the brain signify a great problem for patients. These are the second cause of death in the world [181]. Research shows that these brain-related illnesses especially arise in aged people, signifying that homeostatic reactions that weaken in the elderly are no longer operative. The brain is particularly sensitive to oxidative injury since it has an extraordinary and detailed metabolic action. It is the organ that has the highest consumption of oxygen in the body, high levels of lipids peroxidation, and high amounts of iron, all acting as an oxidant. For this reason, neuronal cells and tissues are extremely vulnerable to metabolic injury and connected OS. Generally, antioxidant usage is not entirely helpful because the harmful effects of OS are life-threatening. Apoptosis is linked with cell injury and possibly with damage to tissue that could be reparable. However, the oncological developments are more dangerous [182].

Aging is the main cause of numerous neurodegenerative disorders. Nrf2 action drops with aging in animals as well as humans due to an escalation in OS, mitochondrial malfunction, and changed gene expression [183]. Many confirmations specify that the lack of an important homeostatic response is clearly associated with Nrf2 absence. Nrf2-knockout animal studies revealed that pathological modifications are observed with aging-like diseases related to neurodegeneration [184,185] that are also observed by aging in humans. Furthermore, it was found that many vital pathways were changed Nrf2-knockout animal brains.

*NFE2L2* is the coding gene of Nrf2 and triggers the expression of over 250 genes. These genes contribute to detoxification systems, redox metabolism, and inflammation reactions in the brain. All these reactions are associated with the formation of neurodegenerative disorders [186]. Nrf2 is a regulator of glucose metabolism in neurons and astrocytes. Using an animal experiment, Esteras et al. [187] showed that the activation of Nrf2 clearly escalates glucose uptake into neurons and astrocytes. A lack of Nrf2 negatively affects neuronal formation and neurons reliant on astrocytic Nrf2 to maintain redox balance and energy homeostasis. Nrf2 regulates the microglial system and could be considered a pharmacological target in some neurodegenerative diseases. In vitro studies revealed that Nrf2 activation prompts the antioxidative response system, diminishes peroxide formation, and modulates phagocytosis of red blood [72,188]. Neurons use Nrf2 to maintain proteostasis. Misfolded protein increase and accumulation tempt excess formation of ROS, which alter redox-sensitive cysteines of KEAP1, causing the discharge, stabilization, and nuclear localization of Nrf2. During aging Nrf2 transcriptional action and capability to maintain proteostasis is reduced, and this is one of the key reasons for the progress of neurodegenerative disorders. It is suggested that the activation of Nrf2 and, subsequently, proteostasis may prevent or suspend the build-up of protein aggregation and cerebrovascular diseases [189].

## 12. Nrf2 and Alzheimer’s Disease

Senile plaques originated via the accumulation of β-amyloid(Aβ) and neurofibrillary tangles yielded by hyperphosphorylation of the tau protein are important pathological features of Alzheimer’s disease (AD) [190]. AD influences more than 50 million people. There are miscellaneous pathogenic hypotheses for AD, such as the cholinergic hypothesis, the Aβ toxicity hypothesis, the tau protein hypothesis, and the inflammation hypothesis, but the pathogenesis of AD still must be clarified [191]. A recent investigation revealed that Chlamydia pneumonia infection is closely related to AD pathogenesis. Tracking the disease, triggered microglia and astrocytes secrete pro-inflammatory cytokines, including IL-1β, TNFα, and IL-6, which are neurotoxic and directly increase Aβ production by activating β-site amyloid-precursor-cleaving enzyme (BACE).

In animal models of AD, Nrf2 inhibits its expression by attaching to AREs in the BACE promoter and inhibits Aβ production. It can also generate nuclear dot protein 52 (NDP52) by binding to AREs in the NDP52 promoter, thereby decreasing p-tau levels in AD [185,192,193]. Thus, the activation of Nrf2 with drug intervention may play a positive role in treating AD patients. Presently, four Nrf2 agonists have entered clinical investigation associated with AD treatment (Table 5).

## 13. Nrf2 and Parkinson’s Disease

Parkinson’s disease (PD) is a chronic progressive nervous system disorder. In late-stage PD, powerful tremors, motor retardation, muscle stiffness, and loss of balance appear [194]. In sporadic and familial PD, α-synuclein(α-syn) aggregates into Lewy bodies and Lewy neurites, which are cytotoxic to dopaminergic neurons and can lead to mitosis and improve mitochondrial autophagy [195]. A boost in dopamine may impact mitochondrial function, improve ROS levels, influence Nrf2 activity, alter the response to antioxidant damage [196,197,198], and promote the progressive production and accumulation of Aβ [199]. These effects lead to dysregulated cellular function. Nonetheless, Nrf2 activation can neutralize ROS, inhibit inflammatory processes, and restore cellular redox equilibrium [200,201,202,203,204]. In PD, there are lowered protein expression levels of phosphatase and tensin homolog (PTEN)-induced kinase (PINK) and Parkin protein; the reductions in these proteins impact mitochondrial function, induce depolarization and fragmentation, and reduce adenosine triphosphate (ATP) concentrations [205]. The Nrf2 upregulation generated by antioxidant treatment was established to improve thioredoxin-1(TrX-1), inhibit the formation of nucleotide-binding domain leucine-rich repeat-related (NLR) family pyrin domain-containing 3 (NLRP3) inflammatory bodies, and enhance neuronal apoptosis in amyloid precursor protein plus presenilin-1 (APP/PS1) mice [206]. Although some mechanisms are not fully understood, Nrf2 can be thought to be a valuable therapeutic target for PD [207]. Four Nrf2 agonists have entered clinical trials for the treatment of PD (Table 6).

**Figure 7:**
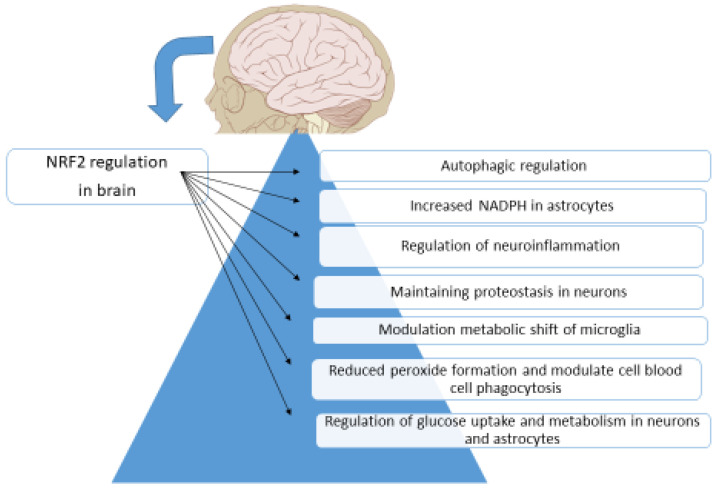
Nrf2 regulation in the brain.

Cerebrovascular disorders are progressively improvable since OS and autophagy have been recognized as significant accomplishments. Recently, it was revealed that there are negative effects of OS and autophagy on cerebrovascular disorders. Animal studies proved that Nrf2 activation plays a defensive role after cerebral ischemia since it acts as a transcription factor against OS and controls the synthesis of ROS scavenger enzymes [208]. Autophagy inhibitors are able to stop the nuclear translocation of Nrf2 and decrease the expression of Nrf2 target genes. This action supports the evidence that autophagy shows antioxidant and neuroprotective roles in brain cells and tissues by stimulating Nrf2 [209]. Animal studies showed that the lack of Nrf2 reduces the inflammatory reaction dramatically in the brain [210]. Therefore, an efficient Nrf2 pathway is vital to control neuroinflammation in response to OS in the brain (Figure 7).

## 14. Future Perspectives

Mitochondrial malfunction and substantial inflammatory reactions are intensely connected with cerebrovascular disorders. OS thus plays a chief role in the pathophysiology of brain-associated diseases. The majority of neurodegenerative disorders are considered by the damage of homeostatic activities that regulate redox metabolism, neuroinflammation, and proteostasis.

Nrf2 is considered a fundamental controller of the antioxidant guard to defend numerous organs from injurious impairment and has been recognized as a favorable drug target for the management of diseases like cancer and autoimmune disorders [211]. The Nrf2-based therapeutic approaches specifically aimed at the management of autoimmune conditions mainly point to molecules able to stimulate the Nrf2 activation pathway by promoting the covalent alteration of cysteine groups in Keap1. Stimulation of Nrf2 could be realized by Bach1 gene knockout, which has revealed defensive activities in diverse disorders, indicating how Bach1 binding molecules could represent a new tool to increase Nrf2 activity that might be beneficial in the regulation of autoimmune conditions [212].

There is some contradiction in the therapy for autoimmune disorders regarding the Nrf2 pathway. It is likely that autoimmune disease therapies would benefit from the activation of Nrf2 since it has antioxidant and anti-inflammatory activities that guard organs and tissues. It is not entirely understood whether the current Nrf2 activators are able to inhibit inflammation and OS in autoimmune conditions.

In consideration of its antioxidant properties and the complex network of pathways in which it is involved, Nrf2 is associated with the occurrence of many disorders. Hence, Nrf2 is thought a specific target for the avoidance and management of countless chronic diseases. The relationship between Nrf2 and disease conditions is multifaceted, so it is important to be cautious while developing therapies. Recently, the activation of Nrf2 had to be successful in experimental models of disorders. However, various cancers and metabolic conditions have persistently raised the amount of Nrf2, representing a necessity for targeted Nrf2 inhibition. We still do not know if specific inhibitors of Nrf2 are synthesized, but we know that targeted inhibitor molecules of Nrf2 are also desirable.

Research indicates a crucial part of Nrf2 in disorders associated with inflammation, cancer, and autoimmune disorders. However, advanced research is essential to define the exact mechanism and behavior of Nrf2 in the immune defense system to develop novel approaches for therapies.

## Figures and Tables

**Table 1:** Inducers of Nrf2 activity [20].

Inducer	Example
Endogenous signaling compounds/metabolites	H_2_O_2_ Lipid peroxidation products Nitric oxide 8-Nitro-cGMP Hydrogen sulfide Methylglyoxal
Oncometabolites	Fumarate Succinylacetone
Immunometabolite	Itaconate
Dietary compounds	Sulforaphane Curcumin
Pharmaceuticals	Dimethyl fumarate Bardoxylone
Microorganisms	Bacteria/lipopolysaccharide Marburg virus *Plasmodium infection*
Extracellular inducers	Heat Laminar flow UVA radiation Exercise

**Table 2:** Nrf2-targeted therapeutics [21].

Therapeutics		Mechanism of Action	Molecules
Electrophilic NRF2 activators	Natural products	Electrophilic modification of KEAP1-C151	Sulphorapane Bixin
	Natural product-derived	Electrophilic modification of KEAP1-C151	Dimethyl fumarate Bardoxolone methyl
Pro-electrophilic NRF2 activators	Natural products	Electrophilic modification of KEAP1-C151	Carnosic acid Carnosol
Non-electrophilic compounds	Peptides	Binding to KEAP1 Kelch domain	Ac-DPETGEL-OH (7mer) FITC-β-DEETGEF-OH (7mer) FITC-LDEETGEFL-NH2 (9mer) FAM-LDEETGELP-OH (9mer)
	Small molecules	Binding to KEAP1 Kelch domain	Compound 2 Cpd15 Cpd16 (SRS)-5 AN-465/144580
KEAP1-independent NRF2 activators	Natural products	GSK-3 inhibition	Nordihydroguaiaretic acid
	Synthetic	HRD1 inhibition	LS-102
NRF2 inhibitors	Natural products	Global protein translation inhibitor	Brusatol

Ac: acetyl, FITC: fluoresceine isothiocyanate, FAM: carboxyfluoresceine.

**Table 3:** Three Nrf2 agonists used in some clinical trials for SLE.

Intervention	Topic	Phase	Trial Country	Primary Endpoints	Dose	Subjects
Curcumin	Effect of curcumin on systemic lupus erythematosus	2	California, United States	Change in SLEDAI.	Intervention: 2 g of curcumin supplement per day.	Sample size: 23; Gender: all; Ages: 18 years and older.
	Vitamin D and curcumin piperine attenuates disease activity and cytokine levels in systemic lupus erythematosus patients	2	Indonesia	1. Disease activity from the SLE patients after the treatments; 2. fatigue assessment from the SLE patients after the treatments; and 3. comparison of cytokine levels before and after the treatments.	The third group received 400 IU cholecalciferol (Nature Plus) t.i.d and curcumin (600 mg)–piperine (15,800 mg) (Bioglan) one time daily.	Sample size: 45; Gender: all; Ages: 18 years to 45 years.
Vitamin D3	Vitamin D3 treatment in pediaric systemic lupus erythematosus	2	California	Change in average IFN module expression level. Percentage of subjects by treatment arm experiencing any adverse event (AE) ≥ grade 3.	Supplementation of 6000 IU vitamin D3 by mouth daily until the subject’s serum 25 (OH) level was ≥40 ng/mL, at which point the supplementation dose was reduced to 4000 IU/day. Note: subjects weighing < 40 kg (kg) at study entry received their dose five days a week, and all other subjects received their dose seven days a week.	Sample size: 7; Gender: all; Ages: 5 years to 20 years.
	Vitamin D3 in systemic lupus erythematosus	2	United States	Percent of patients with an IFN alpha signature response at week 12.	Dose of 8% vitamin D3 powder, 84% microcrystalline cellulose, 8% fumed silica by weight.	Sample size: 57; Gender: all; Ages: 18 years and older.
	Vitamin D to improve endothelial function in SLE	2	United States	Change at week 16 in % flow-mediated dilation in those who did and did not replete vitamin D.	Dose of 5000 international units versus 400 international units as an active comparator.	Sample size: 9; Gender: all; Ages: 18 years and older.
	Vitamin D therapy in patients with systemic lupus erythematosus (SLE)	1	United States	Hypercalcuria.	Cholecalciferol 800 IU oral daily. Cholecalciferol 2000 IU oral daily. Cholecalciferol 4000 IU oral daily.	Sample size: 18; Gender: all; Ages: 18 years to 85 years.
	Vitamin D and curcumin piperine attenuates disease activity and cytokine levels in systemic lupus erythematosus patients	2	Indonesia	1. Disease activity in SLE patients after the treatments; 2. fatigue assessment of SLE patients after the treatments; and 3. comparison of cytokine levels before and after the treatments.	The second group received a tablet containing curcumin (632 mg)–piperine (15,800 mg) (Bioglan) one time daily and a placebo (Saccharum lactis) t.i.d.	Sample size: 45; Gender: all; Ages:18 years to 45 years.
	Effect of vitamin Dsupplement on disease activity in SLE	Not applicable	Thailand	The effect of vitamin D supplementation on SLE disease activity.	Add on vitamin D2 (calciferol) 40,000 IU/wk (2 cap) for 12 weeks.	Sample size: 100; Gender: all; Ages: 18 years and older.
	The effect of vitamin D supplementation on disease activity markers in systemic lupus erythematosus (SLE)	Not applicable	Egypt	Decrease in SLE disease activity.	2000 IU/day for 12 months.	Sample size: 248; Gender: all; Ages: 30 years and older.
SM934	Safety and efficacy of SM934 compared to placebo in adult subjects with active systemic lupus erythematosus	2	China	1. Percentage of subjects with lupus low disease activity score (LLDAS) in each group; 2. percentage of subjects with systemic lupus erythematosus responder index 4 (SRI-4) response in each group; and 3. percentage of subjects with treatment-emergent adverse events (TEAEs) in each group.	SM934 10 mg (five tablets) p.o. qd in combination with steroids.	Sample size: 48; Gender: all; Ages: 30 years and older.

**Table 4:** Two Nrf2 agonists used in some clinical trials for RA [180].

Intervention	Topic	Phase	Trial Country	Primary Endpoints	Dose	Subjects
Dimethyl fumarate	Efficacy and safety study of BG00012 with methotrexate in patients with active rheumatoid arthritis	2	Australia	The primary objective is the proportion of subjects with ACR20 response in their RA at week 12.	Dose of 480 mg/day, oral, and 720 mg/day, oral.	Sample size: 153; Gender: all; Ages: 18 years to 75 years.
Curcumin	Curcuma Longa L in rheumatoid arthritis	1; terminated (insufficient enrollment)	United States	Number of participants with adverse events as a measure of safety and tolerability.	Four 250 mg curcumin capsules twice a day for one month.	Sample size: 3; Gender: all; Ages: 18 years and older.
	Curcumin in rheumatoid arthritis	Early phase 1	United States	American College of Rheumatology 20%. Time frame: 4-month period.	Four capsules once a day for 2 weeks, and then the dose was increased to four capsules twice a day beginning at week 3. Subjects remained at this dose for an additional 13 weeks for a total 16 weeks. After 16 weeks, the same procedures were repeated for another 16 weeks.	Sample size: 40; Gender: all; Ages: 18 years to 75 years.

**Table 5:** Four Nrf2 agonists used in some clinical trials for AD [180].

Intervention	Topic	Phase	Trial Country	Primary Endpoints	Dose	Subjects
EGCG	Prevention of cognitive decline in ApoE4 carriers with subjective cognitive decline after EGCG and a multimodal intervention	N/A	Spain	Preclinical Alzheimer cognitive composite plus exe-like score (ADCS-PACC-like).	Oral 532 mg/day (weight > 50 kg). Oral 266 mg/day (weight < 50 kg).	Sample size: 200. Gender: all. Ages: 60 years to 80 years old.
	Sunphenon EGCg (epigallocatechin-gallate) in the Early stage of Alzheimer’s disease	2/3	Germany	ADAS-COG (score 0–70) (baseline to treatment). Time frame: 18 months.	Months 1–3: 200 mg/day (200-0-0 mg); months 4–6: 400 mg/day (200-0-200 mg); months 7–9: 600 mg/day (400-0–200 mg); and months 10–18: 800 mg/day (400-0-400 mg).	Sample size: 21. Gender: all. Ages: 60 years and older.
	Sunphenon EGCg (epigallocatechin-gallat) in the early stage of Alzheimer’s disease—SUN-AK	2	Germany			Sample size: 50. Gender: all. Ages: 18 years and older.
Sulforaphane	Effects of sulforaphane in patients with prodromal to mild Alzheimer’s disease	N/A	China	The Alzheimer’s Disease Assessment Scale.	Oral 2550 mg once a day for 24 weeks.	Sample size: 160. Gender: all. Ages: 50 years to 75 years old.
Resveratrol	BDPP treatment for mild cognitive impairment (MCI) and prediabetes or type 2 diabetes mellitus (T2DM)	1	United States	Assessment of AEs and SAEs. Brain penetrance of BDPP. Neuropsychiatric Inventory and Cornell Scale for Depression in Dementia. Memory, executive function, and attention measures (composite).	N/A	Sample size: 14. Gender: all. Ages: 50 years to 90 years old.
	Short-term efficacy and safety of perispinal administration of etanercept in mild to moderate Alzheimer’s disease	1	United States	Difference in effects of treatment for 6 weeks with etanercept + nutritional supplements versus nutritional supplements alone on the mini-mental status examination (MMSE) score.	N/A	Sample size: 12. Gender: all. Ages: 60 years to 85 years old.
	Resveratrol for Alzheimer’s disease	2	United States	Number of adverse events. Change from baseline in volumetric magnetic resonance imaging (MRI).	Begin at 500 mg taken once daily and increase after 13 weeks to 1 g taken by mouth twice daily.	Sample size: 119. Gender: all. Ages: 50 years and older.
	Pilot study on the effects of resveratrol supplement in mild to moderate Alzheimer’s disease	3; withdrawn (PI left institution)	United States	Cognition. Time frame: 52 weeks.	Oral 215 mg once a day for 52 weeks.	Sample size: 0.
	Randomized trial of a nutritional supplement in Alzheimer’s disease	3	United States	Alzheimer’s Disease Assessment Scale (ADAScog). Time frame: one year.	N/A	Sample size: 27. Gender: all. Ages: 50 years to 90 years old.
Curcumin	KARVIAH_XTND: Longitudinal follow-up study examining the health and well-being of participants for identifying new biomarkers and the impact of lifestyle (following a 12-month intervention of curcumin for the prevention of Alzheimer’s disease)	N/A	Australia	Blood biomarker compared with brain amyloid levels. Blood biomarkers and PET imaging results.	N/A	Sample size: 100. Gender: all. Ages: 65 years and older.
	Curcumin and yoga therapy for those at risk of Alzheimer’s disease	2	United States	Curcumin effects (first six-month period) or curcumin and aerobic yoga effects (second six-month period) on the changes in the levels of blood biomarkers for mild cognitive impairment relative to baseline or relative to placebo or non-aerobic yoga.	Oral 800 mg curcumin in four capsules BID per day prior to meals.	Sample size: 80. Gender: all. Ages: 50 years to 90 years old.
	KARVIAH Sub-study: Examining the use of curcumin on cognition and mood in an older population	2	Australia	Attention tasks and working memory as measured using a computerized cognitive battery (CogState).	Oral 500 mg three times daily.	Sample size: 40. Gender: all. Ages: 65 years to 90 years old.
	Effect of curcumin (tumeric) in Alzheimer’s disease	N/A	Iran (Islamic Republic of)	MMSE and quality of life questionnaires. Time frame: before and after intervention (12 weeks).	Oral 500 mg twice a day for 12 weeks.	Sample size: 70. Gender: all. Ages: no age limit.
	The epigenetic effect of curcumin as measured in the blood and observed with lifestyle for the prevention of Alzheimer’s disease	2	Australia	Measurement of blood biomarkers within healthy and MCI groups.	Oral 1.5 mg daily (×3 divided doses) for a period of 3 or 6 months.	Sample size: 60. Gender: all. Ages: 65 years to 90 years old.
	McCusker KARVIAH: Curcumin in Alzheimer’s disease prevention	2	Australia	AD-related blood biomarker profiles. Pib PET imaging. Neuropsychological tests. Time frame: up to 12 months.	Dose of 500 mg daily for 2 weeks, progressing to 500 mg twice daily (1000 mg/daily) for 2 weeks, and then 500 mg three times daily (1500 mg) for a period of 12 months in total.	Sample size: 134. Gender: all. Ages: 65 years to 90 years old.
	Biocurcumax from curry spice turmeric in retaining cognitive function	N/A	Australia	Psychometric testing using mini-mental state examination (MMSE), CAMDEX-R, (CAMCOG)-R, etc.	Oral 500 mg three times daily (total 1500 mg/day).	Sample size: 134. Gender: all. Ages: 65 years to 90 years old.
	Efficacy and safety of curcumin formulation in Alzheimer’s disease	2	India	To determine if curcumin formulation affects mental capacity in Alzheimer’s patients based on mental exams.	Oral 2000 mg or 3000 mg daily BID.	Sample size: 26. Gender: all. Ages: 50 years to 80 years old.
	A pilot study on curcumin and ginkgo for treating Alzheimer’s disease	1/2	Hong Kong, China	Change in isoprostane level in plasma. Change in A-beta level in serum.	Oral 1 g/4 g once daily.	Sample size: 36. Gender: all. Ages: 50 years and older.
	Curcumin in patients with mild to moderate Alzheimer’s disease	2	United States	Side effect checklist.	N/A	Sample size: 33. Gender: all. Ages: 50 years and older.

**Table 6:** Four Nrf2 agonists used in some clinical trials for PD.

Intervention	Topic	Phase	Trial Country	Primary Endpoints	Dose	Subjects
Vitamin D3	The effects of vitamin D and bone loss in Parkinson’s disease	2	United States	Direct changes in bone formation and resorption were investigated by measuring serum 25-hydroxyvitamin D [25(OH)D] level, serum parathyroid hormone (PTH) levels, serum osteocalcin, and serum n-telopeptides (N-Tx). Time frame: 12 months.	Dose of 1000 IU/day of vitamin D3.	Sample size: 23; Gender: all; Ages: 18 years and older.
	Clinical effects of vitamin D repletion in patients with Parkinson’s disease	4	United State	Change from baseline visit to 3 months (treatment visit #1) in the TUG, timed walking task (8 m), and UPDRS III subscore. Time frame: 6 months.	Dose of 600 IU vitamin D3 capsule daily.	Sample size: 31; Gender: all; Ages: 18 years to 89 years.
	Twelve weeks of vitamin D supplementation and physical activity in PD patients with DBS	Not Applicable	Poland	The effects of vitamin D supplementation and physical activity on the concentration of vitamin D3 in serum and the evaluation of changes before and after 12 weeks of supplementation and physical activity. Time frame: the outcome was assessed up to 1 year after the last collection of blood.	Dosage based on BMI as follows: for BMI under 25–4000 IU/day, for BMI between 25 and 30–5000 IU/day, and for BMI over 30–6000 IU/day.	Sample size: 72; Gender: all; Ages: 40 years to 90 years.
	Effects of vitamin D in Parkinson’s disease (PD)	2	United States	Change in static balance as recorded using dynamic posturography with the sensory organization test (SOT 1–3).	Drug: vitamin D3 Vitamin D3 at 10,000 IU a day. Dietary supplement: calcium Calcium at 1000 mg a day.	Sample size: 101; Gender: all; Ages: 50 years to 99 years.
Resveratrol	Tolerability, safety, and pharmacokinetics of four single doses of BIA 6-512 (Trans-resveratrol) and their effect on the levodopa pharmacokinetics	1	Portugal	1. Maximum observed plasma drug concentration (Cmax) post-dose—levodopa. Time of occurrence of Cmax (tmax)—levodopa. 2. Area under the plasma concentration–time curve (AUC) from time zero to the last sampling time at which concentrations were at or above the limit of quantification (AUC0-t), calculated using the linear trapezoidal rule—evodopa. 3. Area under the plasma concentration versus time curve from time zero to infinity (AUC0-∞), calculated from AUC0-t + (Clast/λz), where Clast is the last quantifiable concentration and λz is the apparent terminal rate constant—levodopa. 4. Apparent terminal half-life, calculated from ln 2/λz (t1/2)—levodopa. 5. Maximum observed plasma drug concentration (Cmax) post-dose—BIA 6-512. 6. Time of occurrence of Cmax (tmax)—BIA 6-512. 7. Area under the plasma concentration–time curve (AUC) from time zero to the last sampling time at which concentrations were at or above the limit of quantification (AUC0-t), calculated using the linear trapezoidal rule—BIA 6-512. 8. Area under the plasma concentration versus time curve from time zero to infinity (AUC0-∞), calculated from AUC0-t + (Clast/λz), where Clast is the last quantifiable concentration and λz the apparent terminal rate constant—BIA 6-512. 9. Apparent terminal half-life calculated from ln 2/λz (t1/2)—BIA 6-512.	One capsule of Madopar^®^ HBS 125 ROCHE, Basel, Switzerland (levodopa 100 mg/benserazide 25 mg) in an open label manner, concomitantly with BIA 6-512/placebo.	Sample size: 20; Gender: all; Ages: 18 years to 45 years.
	Effect of BIA 6-512 at steady-state on levodopa pharmacokinetics with a single dose of levodopa/benserazide 200/50 mg or with a single dose of levodopa/benserazide 200/50 mg plus a single-dose of nebicapone 150 mg	1	Portugal	1. Day 4—Maximum observed plasma drug concentration (Cmax). 2. Day 4—Time of occurrence of Cmax (tmax). 3. Day 4—Area under the plasma concentration–time curve (AUC) from time zero to the last sampling time at which concentrations were at or above the limit of quantification (AUC0-t). 4. Day 4—AUC from time zero to 8 h post-dose (AUC0-τ). 5. Day 4—Area under the plasma concentration versus time curve from time zero to infinity (AUC0-∞). 6. Day 4—Apparent terminal elimination half-life, calculated from ln 2/λz (t1/2). 7. Day 5—Maximum observed plasma drug concentration (Cmax). 8. Day 5—Time of occurrence of Cmax (tmax). 9. Day 5—Area under the plasma concentration–time curve (AUC) from time zero to the last sampling time at which concentrations were at or above the limit of quantification (AUC0-t). 10. Day 5—AUC from time zero to 8 h post-dose (AUC0-τ). 11. Day 5—Area under the plasma concentration versus time curve from time zero to infinity (AUC0-∞). 12. Day 5—Apparent terminal elimination half-life, calculated from ln 2/λz (t1/2).	The investigational products consisted of capsules containing BIA 6-512 25 mg, 50 mg, 75 mg, and 100 mg. Oral doses with 240 mL of potable water.	Sample size: 38; Gender: all; Ages: 18 years to 45 years.
EGCG	Efficacy and safety of green tea polyphenol in de novo Parkinson’s disease patients	2	China	Delay of progression of motor dysfunction.	N/A	Sample size: 480; Gender: all; Ages: 30 years and older.
Sulforaphane	A 6-month study to evaluate sulforaphane effects in PD patients	2	China	Cognitive improvement assessed using the MATRICS Consensus Cognitive Battery (MCCB) composite score.	N/A	Sample size: 100; Gender: all; Ages: 40 years to 75 years.

## Data Availability

This review article has no research data to be shared.
